# On the Nature of the Gastric Juice, and the Changes It Effects on Nitrogenized and Unnitrogenized Food

**Published:** 1850-03

**Authors:** H. Bence Jones

**Affiliations:** M. D., A. M. Cantab., F. R. S., Physician to St. George’s Hospital


*On the Nature of the Gastric Juice, and the Changes it Effects on Nitrogenized and Unnitrogenized Food.* By H. Bence Jones, M. D., A. M. Cantab., F. R. S., Physician to St. George's Hospital.—On the 5th of March, 1849, breakfast having been taken at eight A. M., on boiled beef, bread, and spring water, at 9.45, by position and voluntary effort, about a pint of substance was ejected. The first ounces were scarcely acid to the taste; the last portion was most intensely acid. The whole quantity was thrown on a filter, and a clear, yellowish-brown liquid passed through. This clear liquid was intensely acid to test paper. It coagulated slightly on the addition of nitric acid and heat. The cold acid caused a deepish yellow color and a coagulum, which appeared partly to be soluble by heat, and precipitable by cooling. The specific gravity = 1008.2. 504.1 grains, evaporated in vacuo over sulphuric acid, evolved a small quantity of gas.

Residue + basin = 549.3 grains.
basin = 505.0 "

March 15th.—Residue ... = 44.3 grs. = 8.8 per cent.

The residue was yellowish, semi-transparent, took the impression of the nail, smelt of musk and sugar! ! When mixed with cold distilled water, it became ropy, and very adhesive. The solution was highly acid. The taste was sweet, acid, bitter, nauseous. A few drops of the solution, with sulphate of copper and liquor potassæ, gave an intense blue, with a trace of purple; and the reduction of the oxide of copper was very rapid, excessive, and red.

In this bottle you see this gastric juice. Here is its strong acid reaction on litmus, and here you see the rapid reduction of the oxide of copper. The food being bread, it is probable that some of the sugar was formed in the process of fermentation, and not produced altogether in the stomach.

This gastric juice, then, is a highly acid fluid secreted by the stomach. It consists—1, of water and free acid; 2, of salts; 3, of non-nitrogenous organic substances; 4, of albuminous or nitrogenized substance. Of these substances, the most important is the free acid. What acid, it has not yet been determined. Hydrochloric, phosphoric, acetic, lactic, and butyric acids have each been said to exist in the gastric juice. The hydrochloric and phosphoric are mineral or inorganic acids. The rest are organic acids,—possibly arising from starch, sugar or fat, or other components of the non-nitrogenized part of our food. Thus these organic acids might be formed; but whence can the inorganic acids come? Hydrochloric and phosphoric acids exist only in the food and blood, combined with soda, potash, or lime; as in common salt, phosphate of soda, or phosphate of lime. To set free the acids, the alkalies must be separated. If one equivalent of hydrochloric acid is set free in the stomach, one equivalent of soda must be set free in the blood. The greater the quantity of acid in the stomach, the greater the quantity of alkali in the blood, and the more alkaline the serum must become. Whether this is effected by galvanic action, nervous action, or muscular action, is at present altogether unknown. Those who say anything, say it is by vital action. Whatever may be the nature or seat of the decomposing force,—whether galvanic, nervous, or muscular,—whether in the cells of the epithelium of the stomach-tubes, or in the muscular structure,—we cannot admit that inorganic acid can be poured into the stomach without an equivalent quantity of alkali being set free in the blood, when digestion is completed, the acid is re-absorbed with the food, and the alkalescence of the blood must be altered in the opposite direction. When the stomach is empty, there is little, if any acid there then. When food is taken, the quantity of acid begins to increase, and gradually reaches the greatest amount poured out; and then by absorption, or by escape through the pylorus, the quantity of acid begins to decrease, until the stomach is again empty.

For the purpose of keeping up a constant supply of at least one inorganic acid, man has been led at all times, and in all circumstances, to seek for salt, as necessary to his existence. What the influence of chloride of sodium undecomposed, and of other salts, is on the solubility of albumen or starch, has not yet been sufficiently determined.

The next most important constituent of the gastric juice, after water and acid, is the albuminous substance. Its exact nature is not known. I cannot consider it as epithelium. It is far more likely to be a substance like diastase; not albumen, not epithelium, but a peculiar albuminous substance. Its exact composition is not known; but probably it is a substance undergoing changes which it can communicate to other contiguous substances. It was precipitated by a weak alcohol from infusion of pig's stomach, by Wasman, and he called it pepsin; it requires re-examination.

The non-albuminous organic substances in the gastric juice, are the organic acids and fatty substance; the latter probably exists in a very small quantity. This complex gastric juice cannot act precisely in the same way on two classes of substances so very different as nitrogenized and non-nitrogenized food.

Firstly. *The action of the Gastric Juice on the Nitrogenized substances in the Food.*—The fine state of division of the food, the smallness of its amount, the constant muscular motion of the stomach, and the temperature of the body—these all assist the solution of the fibrin, albumen, or casein. Strong acids very easily dissolve these albuminous substances, and the dilute acid of the stomach, in consequence, perhaps, of some influence of the nitrogenized pepsin, or animal diastase (?), is made to act as energetically on the albuminous food, as strong acid would do. In this action there is nothing vital; it takes place as well out of the body as in it. The elements of the albumen cannot be converted so as to form water, salts, sugar or fat. There is no formation of incipient albumen. If you please to call solution, reduction or combination of water with the substance dissolved, you may say the albumen is reduced; to me it is far more simple and quite as comprehensible to speak of it as a process of solution, and as nothing else.

Secondly. *On the Action of the Gastric Juice on Non-Nitrogenized Food, Starch, Sugar, Fat, &c.*—Starch is perfectly insoluble in water and in dilute acids, but by the action of dilute acid, it easily undergoes a change, by which it is rendered soluble. The action of strong sulphuric acid on starch, and the formation of sugar thereby, is probably well known to you already; but there is no strong acid in the stomach.

The relation of starch to British gum, or dextrine, or, as it has been called, soluble starch, is also well known to you. There are many ways of changing the insoluble starch into soluble dextrine. One very perfect method has been practiced in France, of treating the starch, at the temperature of 100° Fahr., with dilute hydrochloric or oxalic acid, and thus dextrine is readily formed. There is no doubt that the temperature and dilute hydrochloric acid in the stomach effect the same conversion as you see has been effected in this flask; further action of the acid and heat, converts the dextrine into sugar. It has been said, that the starch is acted on by the alkaline saliva, but directly the saliva reaches the stomach, it must be neutralized by the gastric juice.

*On the Action of the Gastric Juice on Sugar.*—The ready solubility of sugar in water requires no illustration. A portion of the sugar which is taken as such, or is formed from starch in the stomach, without doubt passes into the blood as sugar; but it appears to me highly probable, from Fremy's experiments, that a portion is changed into some of the vegetable acids. The acetic and lactic acids may thus be formed, and these, in part, perhaps may become lactates and acetates of soda in the blood. And we know, from direct experiment, what happens to vegetable acid salts injected into the blood, or taken into the stomach; they are oxydized or burnt, giving heat and carbonic acid salts, which pass off in the urine. All free vegetable acids are probably changed partly into carbonic acid in the blood. Thus, then, probably the progress of a grain of starch may be traced. It forms, first, dextrine; secondly, sugar; thirdly, vegetable acid; fourthly, carbonic acid.

The ascending conversion of sugar into albumen cannot be admitted until it is proved. There is not an experiment which renders such a change probable.

The descending conversion of sugar into fat, a substance also containing no nitrogen,—that is, of one kind of non-nitrogenized substance into another kind belonging to the same class, is most fully proved. Bees fed on crystallized sugar made wax, and animals form more fat than the food they are fed with contains; but this fat is not formed in the stomach, and therefore does not concern us now. The changes take place in the minute textures of the body, and not in the stomach.

*On the Action of the Gastric Juice on Fat and Oil.*—How is fat made soluble at the temperature of the body? Is oil absorbed? The following is an experiment by Tiedemann and Gmelin. A dog was fed for four days on butter; three hours after the last meal he was killed.

*a.* Stomach, contained butter, and the contents were very acid. *b.* Small intestine contained butter and bile, and was acid strongly. *c.* The cæcum contained butter. *d.* The rectum contained butter. *e.* The chyle was very milky, and cleared with ether. *f.* The blood of the vena cava inferior contained much fat. *g.* The urine was thick; filtered, butter soluble in alcohol was left on the filter. They add: "One of our pupils, who likes fat, has frequently found it in his urine." This has also sometimes been observed after cod-liver oil. Oil and albumen form an emulsion slightly soluble in water; but the pancreatic fluid and the bile are generally considered as the agents which make the fat and oil of the food soluble.

Lastly, the phosphates and sulphates of soda, chloride of sodium, are soluble in water. The earthy phosphates are dissolved by the hydrochloric acid. Even silica, in minute quantity, is contained, dissolved in water, and hence these salts pass into the blood.

The formation of the gastric juice is certainly no chemical process, but its action is entirely chemical, though it is aided by the motion which the muscular coat of the stomach produces; and muscular contraction is as distinct as sensation from chemical action. Nor can the absorption of the dissolved substances be altogether considered as a vital action. It is certainly subject to the laws of the diffusion of one liquid into another, to the laws of endosmosis, or diffusion through a membrane, and to capillary action. This subject, alone, might occupy me for many lectures; it belongs more to my colleague, the lecturer on physiology, than to me. I pass on, therefore, to the next subject, the formation of blood from the absorbed food, the assimilation of the food to the blood, How is it that the blood keeps its composition? The food is dissolved. and then absorbed, and the blood somehow makes it into blood; somehow preserves its own composition. What, then, is this blood, chemically? how does it differ from food in composition?—*Lon. Lancet.*

